# 

**Published:** 1860-09

**Authors:** 


### LIST OF COLLABORATORS.

ARMOR, S. G., M.D.,
Late Professor of Pathology and Clinical Medicine, Missouri Medical College.

ATKINSON, WM. B., M.D.,
Lecturer on Obstetrics, and Diseases of Women and Children, Philadelphia.

ATLEE, JOHN L., M.D.,
Late President Pennsylvania State Medical Society.

ATLEE, WALTER F., M.D.,
Philadelphia.

BARCLAY, ROBERT G., M.D.,
Jerusalem, Syria.

BELL, JOHN, M D.,
Philadelphia, formerly Professor of Medicine, Medical College of Ohio.

BEMISS, S. M., M.D.,
Professor of Clinical Medicine, University of Louisville.

BLACKBURN, L. P., M.D.,
Natchez, Miss.

BLACKIE, GEORGE C., M.D.,
Professor of Botany, University of Nashville.

BLACKMAN, G. C., M.D.,
Professor of Surgery, Medical College of Ohio.

BOBBS, J. L., M.D.,
Formerly Professor of Anatomy, Indianapolis.

BOWEN, W. S., M.D.,
New York.

BOZEMAN, N., M.D.,
Montgomery, Ala.

BRADFORD, J. T., M.D.,
Augusta, Ky.

BRINTON, JOHN H., M.D.,
Lecturer on Operative Surgery, Philadelphia.

BUMSTEAD, FREEMAN J., M.D.,
New York.

CARNOCHAN, J. M., M.D.,
Professor of Surgery in the New York Medical College.

CHIPLEY, W. S., M.D.,
Professor of Medicine, Transylvania University.

CLAPP, A., M.D.,
New Albany, Indiana.

CLIFFE, D. B., M.D.,
Franklin, Tenn.

DA COSTA, J., M.D.,
Lecturer on the Practice of Medicine at the Philadelphia Medical Association.

DICKSON, SAMUEL H., M.D.,
Prof. of Medicine, Jefferson Medical College.

DUNGLISON, RICHARD J., M.D.,
Physician to the Burd Orphan Asylum, and to the Albion Society, Philadelphia.

DUPIERRIS, M., M.D.,
Havana, Cuba.

EDGAR, W. F., M.D.,
United States Army.

FARRAR, S. C., M.D.,
Jackson, Miss.

FENNER, C. S., M.D.,
Memphis, Tenn.

FISCHER, EMIL, M.D.,
Philadelphia.

FLINT, AUSTIN, M.D.,
Professor of Clinical Medicine, Auscultation, and Percussion, New Orleans School of Medicine.

GADBURY, W. Y., M.D.,
Benton, Miss.

GIBBS, O. C., M.D.,

Chautauque County, New York.

HALL, J. P., M.D.,
Glasgow, Ky.

HAMMOND, WM. A., M.D.,
United States Army.

HARDIN, JOHN, M.D.,
Professor of Obstetrics, Kentucky School of Medicine, Louisville.

HARTSHORNE, EDWARD, M.D.,
Surgeon to the Pennsylvania Hospital.

HASKINS, E. B., M.D.,
Professor of Medicine, Shelby Medical College, Nashville, Tenn.

HENTZ, C. A., M.D.,
Marianna, Florida.

HEWSON, ADDINELL, M.D.,
One of the Surgeons to Wills Hospital for Diseases of the Eye, Philadelphia.

HOLLINGSWORTH, SAMUEL L., M.D.,
Formerly Editor of the Medical Examiner, Philadelphia.

JEWELL, WILSON, M.D.,
Philadelphia, formerly President of the Quarantine and Sanitary Convention.

KEATING, WM. V., M.D.,
Lecturer on Obstetrics and the Diseases of Women, at the Phila. Medical Association.

KERR, JOHN G., M.D.,
Canton, China.

KUNKLER, G. A., M.D.,
Madison, Ia.

LOGAN, BEN., M.D.,
Shreveport, La.

LOPEZ, A., M.D.,
Mobile, Ala., formerly President of the Alabama State Medical Society.

MEIGS, J. AITKEN, M.D.,
Professor of the Institutes of Medicine in the Medical Department of Penna. College.

MITCHELL, S. WEIR, M.D.,
Lecturer on Physiology at the Philadelphia Medical Association.

MOREHOUSE, GEORGE R., M.D.,
Philadelphia.

PACKARD, JOHN H., M.D.,
Philadelphia.

RAPHAEL, B. I., M.D.,
New York.

REEVE, J. C., M.D.,
Dayton, Ohio,

REEVES, JAMES E., M.D.,
Fairmount, Virginia.

RIVES, L. C., M.D.,
Formerly Professor of Midwifery, Medical College of Ohio.

SMITH, J. LAWRENCE, M.D.,
Professor of Chemistry, University of Louisville.

SNEED, W. C., M.D.,
Frankfort, Ky., formerly President of Kentucky State Medical Society.

SPILMAN, C. H., M.D.,
Harrodsburg, Ky., formerly President of Kentucky State Medical Society.

STORER, HORATIO R., M.D.,
Formerly one of the Physicians to the Boston Lying-in Hospital, Boston, Mass.

SUTTON, GEO., M.D.,
Aurora, Ia.

SUTTON, W. L., M.D.,
Georgetown, Ky., formerly President Kentucky State Medical Society.

THOMPSON, S., M.D.,
Albion, Ill., formerly President of the Illinois State Medical Society.

TODD, L. BEECHER, M.D.,
Lexington, Kentucky.

WILLIAMS, E., M.D.,
Cincinnati, Ohio.

### Bi-Monthly Bibliographical Record.

### AMERICAN.

Electro-Physiology and Electro-Therapeutics; showing the best methods for the Medical Uses of Electricity. By A. C. Garratt. 8vo., pp. 708. Ticknor & Fields: Boston, 1860. (From the author.)

Report of Professor Valentine Mott's Surgical Cliniques in the University of New York—Session 1859–60. By S. W. Francis, M.D. 12mo., pp. 209. S. S. & W. Wood: New York, 1860. (From the author.)

A Concise Description of the Anatomy of the Fifth Pair of Nerves: being a Key to the Plate on that Subject. By H. A. Daniels, M.D. pp. 15. Jones & White: Philadelphia, 1860. (From the publishers.)

Proceedings of the Sixty-eighth Annual Convention of the Connecticut Medical Society, held at Hartford, May 23d and 24th, 1860. 8vo., pp. 72. (From the Secretary.)

Statistics of Insanity in the United States. By R. J. Dunglison, M.D. Reprint. 8vo., pp. 40. Philadelphia: J. B. Lippincott & Co., 1860. (From the author.)

Currents and Counter-Currents in Medical Science. An Address delivered before the Massachusetts Medical Society, May 30th, 1860. By Oliver Wendell Holmes, M.D. 8vo., pp. 48. Boston: Ticknor & Fields, 1860. (From the author.)

Proceedings of the Academy of Natural Sciences of Philadelphia. 8vo., pp. 193–284. Philadelphia, 1860. (From the Secretary.)

Annual Address delivered before the Medical Society of Clinton County, New York, June 6th, 1860. By F. J. d'Avignon, M.D. Pamphlet. pp. 18. (From the author.)

On Pleuro-Pneumonia. Evidence before the Commonwealth of Massachusetts pp. 279. 1860. (From Dr. Bigelow.)

The Anatomy and Physiology of the Placenta; the Connection of the Nervous Centres of Animal and Organic Life. By John D. Reilly, M.D., etc. 8vo., pp. 111. New York, 1860. (From the author.)

### FOREIGN.

On Obscure Diseases of the Brain, and Disorders of the Mind: their incipient Symptoms, Pathology, Diagnosis, Treatment, and Prophylaxis. By Forbes Winslow, M.D., etc. 8vo., pp. 576. Blanchard & Lea: Philadelphia, 1860. (From the publishers.)

De la Circulation du Sang dans les Membres et dans la Tête chez l'Homme. Par J. P. Sucquet, M.D., etc. 8vo., pp. 55. J. B. Baillière et Fils: Paris, 1860. (From the author.)

Exposition of a Method of preserving Vaccine Lymph fluid and active; with Hints for the more efficient performance of Public Vaccination. By Wm. Husband, M.D., etc. 12mo., pp. 45. London: Churchill, 1860. (From the author.)

Notes on Nursing: what it is, and what it is not. By Florence Nightingale. Demi 8vo., pp. 140. New York: D. Appleton & Co., 1860. (From S. Hazard.)

The Medical Knowledge of Shakspeare. By J. C. Bucknill, M.D., etc. 8vo., pp. 292. London: Longman & Co., 1860.

Formules Favorites des Practiciens Américains Vivants les Plus Distingués. Traduit de l'Anglais par L. Noirot. 8vo., pp. 234. Paris: Victor Masson, 1860.

Illustrations of Puerperal Fever. By Edward Copeman, M.D., etc. 8vo., pp. 137. London: Churchill, 1860.

Des Tumeurs Fibreuses de l'Utérus. Par Dr. Felix Guyon. 8vo., pp. 139. Paris: Adrien Delahaye, 1860.

Manual of Botany. By John Hutton Balfour, M.D., etc. 8vo., pp. 700. Edinburgh: A. & C. Black, 1860.

Small-pox and Vaccination Historically and Medically Considered. By Alfred Collinson, M.D. 8vo. London: Hatchard & Co., 1860.

Traité des Tumeurs de l'Orbite. Par M. Démarquay. 8vo., pp. 584. Paris: Victor Masson, 1860.

Des Lésions Traumatiques de l'Encéphale. Par Dr. Bauchet. 8vo., pp. 200. Paris: Adrien Delahaye, 1860.

